# Exploring the Shift From HIV Pre-exposure Prophylaxis Awareness to Uptake Among Young Gay and Bisexual Men

**DOI:** 10.3389/fpubh.2021.677716

**Published:** 2021-12-07

**Authors:** Kimberly A. Koester, Xavier A. Erguera, Ifeoma Udoh, Mi-Suk Kang Dufour, Jeffrey H. Burack, Janet J. Myers

**Affiliations:** ^1^Center for AIDS Prevention Studies, University of California, San Francisco, San Francisco, CA, United States; ^2^Pangaea Global AIDS, Oakland, CA, United States; ^3^East Bay AIDS Center, Alta Bates Summit Medical Center, Oakland, CA, United States

**Keywords:** HIV prevention, PrEP uptake, PrEP education, pre-exposure prophylaxis (PrEP), PrEP awareness, PrEP decision-making, qualitative research, youth

## Abstract

**Introduction:** HIV pre-exposure prophylaxis (PrEP) in the form of a daily oral medication is highly effective at preventing HIV. In the United States, awareness about PrEP has steadily increased over time among individuals vulnerable to HIV, however awareness has not translated into widescale uptake. Estimates are that fewer than 20% of 1.2 million Americans for whom PrEP is indicated are utilizing it. We sought to understand how individuals moved from PrEP awareness to PrEP utilization.

**Methods:** We conducted a series (*n* = 31) of in-depth interviews with young people, predominantly gay and bisexual men, ages 18–29 years old between February 2015 and January 2016, as part of the evaluation of a multi-year demonstration project funded to test innovative approaches to improve sexual health outcomes and curb the HIV epidemic in California. Interviews were audio-recorded and transcribed verbatim. We conducted a thematic analysis.

**Results:** We present a continuum of PrEP awareness that spans three phases—basic, moderate and advanced. Participants rarely reported becoming well-informed about PrEP over the course of an initial exposure to PrEP information. Learning occurred after multiple exposures to PrEP information through numerous intersecting forms, messengers and formal and informal communication channels. Positively framed messages delivered by formal messengers emphasizing PrEP as a sensible HIV prevention strategy and explicitly communicating a regard for sexual wellness were overwhelmingly persuasive and facilitated movement to the advanced awareness phase. Once participants reached the advanced phase of PrEP awareness, uptake was possible.

**Conclusions:** Our analysis provides insights into how PrEP awareness led to PrEP uptake among young gay and bi-sexual men. Building demand among those in the basic awareness phase took longer than those in the moderate phase. Individuals involved in formal and informal PrEP education can set reasonable expectations about whether, when and how eventual uptake may occur when keeping the continuum of PrEP awareness framework in mind. Many young, gay and bi-sexual male prospective PrEP users will benefit from positively framed messages that emphasize personal well-being, including social, sexual and emotional benefits of PrEP use.

## Introduction

HIV disease continues to be a serious public health challenge. Globally, 38 million people are living with HIV (1) and ~2 million people become infected with HIV every year (2). Conversely, advances in prevention and treatment coupled with a global commitment to end the epidemic have renewed our optimism for a future in which HIV infection is a rare occurrence. Pre-exposure prophylaxis (PrEP) in the form of a daily oral medication is highly effective at preventing HIV and is typically prescribed as part of a comprehensive package of services that includes routine HIV testing, counseling, and treatment for sexually transmitted infections as needed (3, 4). HIV PrEP has been characterized as a revolution in HIV prevention and has the potential to advance the basic human rights of individuals through higher standards of sexual health (5, 6).

Despite apparent benefits, implementation of PrEP services in the “real world” has been met with slower than desired rates of adoption. In the United States, recent studies estimate that over a quarter of a million people have ever been on PrEP (7) and ~200,000–205,000 are active consumers (8). While these estimates represent a significant increase in utilization of PrEP in the US, current levels of PrEP coverage represent only a fraction of the estimated 1.2 million people for whom it is indicated and who could potentially benefit from this prevention tool (9). With 40% coverage among those meeting at-risk criteria and 62% adhering to PrEP, one third of new infections in the US could be prevented over the next 10 years (10).

Equally concerning is emerging evidence suggesting widening disparities in PrEP access. Individuals filling PrEP prescriptions are more likely to identify as male and white (11, 12); other correlates include over 24 years of age, higher income, and access to health insurance (13). There are also geographic disparities, with nearly 50% of PrEP users located in just five states: New York, California, Florida, Texas, and Illinois (13). There exists a critical need for interventions to address the array of factors that prevent equitable PrEP access and benefit to populations most at-risk for HIV infection.

Lack of awareness of and willingness to use PrEP remain obstinate barriers to widespread uptake (14, 15). Vulnerable populations such as young people, Black and Latino gay and bi-sexual men (GBM) and transgender individuals who could benefit from PrEP may not know about it or know enough about it to seek a prescription (16–18). Perez-Figueroa and colleagues found that among youth and young adults PrEP knowledge was often incomplete—they didn't fully understand “what PrEP is, how it acts to prevent HIV acquisition, and its potential short- and long-term side effects (19).” Olansky and colleagues similarly found that, among Black and Latino GBM, conspiracy belief was inversely related to PrEP knowledge—those who reported conspiracy beliefs were less likely to be aware of PrEP (20). Insufficient knowledge and misrepresentation of PrEP and can seriously hinder its ability to impact the HIV epidemic among vulnerable populations.

Even in states with reported high number of PrEP users—New York and California –disparities between those with and without awareness of PrEP exist (19, 21). For those with some PrEP awareness, knowledge of its existence does not immediately translate into opting to use PrEP. The shift from awareness to talking with a health care provider about PrEP is fraught with challenges. Bauermeister and colleagues noted low levels of awareness of PrEP and lower likelihood of use among youth and young adults due to the lack of insurance, fear of side effects, and medication burden (22). Similarly, a study by Garcia and colleagues found that cost, insurance issues, follow-up medical visits, PrEP related stigmas, access to knowledgeable health care providers, and mistrust of the government as well as health care providers hindered PrEP adoption among Latino MSM (23). Marcus et al. found that only 30% of people recently diagnosed with HIV in northern California had previously discussed PrEP with a provider, a disparity likely to be exacerbated in communities with less access to sexual health services and education (24).

Addressing disparities in PrEP utilization requires a nuanced investigation of the influential social, interpersonal and individual-level barriers and facilitators associated with each stage of the continuum of effective PrEP use from awareness to uptake to adherence (25–27). In this article, we offer insights into the phases of PrEP awareness and uptake, based on qualitative interviews conducted with a sample of ethnically diverse young people, primarily gay and bi-sexual men, participating in a PrEP demonstration project in California, United States. Our study sought to understand the processes associated with how individuals learned about PrEP and how they decided whether it was right for them. Understanding better how young people learned about and made decisions about PrEP has implications for future program implementation, outreach and community education efforts.

## Methods

### Setting

We conducted a series (*n* = 39) of in-depth interviews with young people ages 18–29 years old between February 2015 and January 2016, as part of the evaluation of a multi-year demonstration project funded to test innovative approaches to improve sexual health outcomes and curb the HIV epidemic in California (28). Connecting Resources for Urban Sexual Health, or CRUSH, served people at high risk for HIV infection by expanding access to PrEP and other sexual health services within an existing HIV-primary care clinic located in the eastern region of the of the San Francisco Bay Area (East Bay). The newly established sexual health center provided client-centered, sex positive sexual health services to a population of young gay and other men who have sex with men in a geographic location where comprehensive HIV prevention and sexual health services, including the provision of pre-exposure prophylaxis (PrEP), were not readily available.

### Sampling and Recruitment

In-depth interview study participants were identified and selected from the cohort of youth who independently elected to use PrEP while enrolled in the CRUSH demonstration study. The CRUSH project eligibility criteria have been described elsewhere (3) but briefly included the following: ages 18 to 29, able to provide consent to participate in a research study in English or Spanish, and self-identified as “at-risk” for HIV infection. We employed a purposive sampling strategy with the intention of capturing a wide range of perspectives and experiences (29). To identify our participants, clinic staff who conducted the initial intake, as well as the study coordinators who facilitated the informed consent and conducted the baseline and follow-up assessments were asked to recommend individuals to invite to participate in an in-depth interview(s). Recommendations were vetted by first (KK) and second (XE) authors. Participation in the qualitative study was not contingent on actual use of CRUSH services. Rather, eligibility criteria were designed to be inclusive of participants at all levels of engagement in the CRUSH project. Although there were no specific racial/ethnic inclusion criteria, we oversampled African American and Latino men. The research study was available in English and Spanish, though no one expressed a preference to conduct the interview(s) in Spanish. The University of California San Francisco and Sutter Health Institutional Review Boards approved all procedures associated with the study.

### Interview Guide Development

Our study design included conducting in-depth interviews at two time points (baseline and follow-up). This allowed us to understand experiences related to initial interest and early experiences with PrEP use, as well as subsequent experiences and plans for the future. The two open-ended interview guides were designed to elicit free flowing narratives related to the key areas of interest. The baseline guide focused on exploring individual's experiences becoming socialized around human sexuality, their own sexual awakening, experiences with sexual health education and seeking sexual health services in the region, and describing in-depth their reactions, attitudes and experiences learning about and making the decision that PrEP was right for them. The follow-up guide focused greater attention on social and sexual consequences of PrEP use, as well as documenting in detail individuals' future plans regarding PrEP. These research topics were presented to and vetted by a multi-disciplinary team of clinicians, researchers and community stakeholders to assess the appropriateness and priority of the research topics. Afterwards, the project's community advisory board, composed of consumers and service providers themselves drawn from the local community of youth at risk for HIV, also evaluated and provided essential feedback on the development of the interview guide. We pilot tested the guides with three young people who had previously participated in the formative phase of the CRUSH project.

### Data Collection Procedures

Individuals identified as potential participants were initially approached by the clinic staff prior to one of their study visits to inform them briefly about the opportunity to participate in qualitative interviews. If a participant expressed interest in learning more, they were connected with one of the study coordinators (XE) who provided the prospective informant with a more detailed explanation of the qualitative evaluation activities and answered any immediate concerns. If they provided assent, we proceeded with scheduling the qualitative interview at a mutually agreeable time. A subset of individuals deemed to be particularly knowledgeable and/or articulate about an issue of interest were invited to participate in up to two additional interviews. The interviews were conducted by expert qualitative researchers (KK and XE). The interviews took place in person, in a private space at the CRUSH project site, lasted between 60 and 120 min and were audio-recorded. Prior to initiating the interview, participants were provided with an information sheet that reviewed all compulsory study information (purpose, benefits, risk, alternatives, compensation and study contact information). Individuals were allowed sufficient time to review the information sheet and make an informed decision regarding their participation. After obtaining informed consent, we asked a series of open-ended questions, probing for further detail as needed. We made spontaneous modifications when appropriate e.g., dropping questions that were not applicable. Following the interview, the participants were asked to complete a short demographic questionnaire. Participants were compensated $40 in cash per interview.

### Analyses

We conducted a thematic analysis (30), a multi-step process that included reading and re-reading the data, applying a coding scheme which consisted of both inductive and a priori codes, code interpretation, theme identification, generating tables in order to compare narratives, and vetting findings with stakeholders and community members. All interviews were digitally recorded, professionally transcribed verbatim, de-identified and uploaded into Dedoose, a web-based software used to facilitate the organization and analysis of qualitative data (Version 5.0.11, 2014). KK and XE conducted the coding, code interpretation and theme identification during analysis meetings. We generated a total of 28 codes. For this analysis, we present the themes associated with the following codes: *learning about PrEP* and *motivations to use PrEP*. We distilled salient narratives into tables and identified patterns across the cases. We identified sources of PrEP information, identified contextual factors that influenced receptivity to and the usefulness of information, and mapped participant's trajectories from initial awareness to deciding to uptake PrEP. Once we constructed our themes, we presented our initial findings to an audience of community members [this is also known as a “member check (31)”] to ensure our interpretation of the data were accurate. The member-check included a review of the findings with participants and the members of the study's community advisory board.

## Findings

A total of 45 young people participated in qualitative evaluation activities; multiple interviews were conducted with eight of these informants for a total of 53 interviews. Of these, 31 elected to use PrEP and all but the two cases of heterosexual young women are featured in this analysis. The demographics of the cohort are represented in Table 1.

**Table 1:** Demographics of Study Sample.

**Variables**	**Categories**	***N***	**Mean/range**
Age	Eligible age (18–30yrs)	31	25.06/19–29
Sex	Male	29	93.50%
	Female	2	6.50%
	Transgender	0	0.00%
Race/ethnicity	Black	7	22.58%
	Latino	10	32.26%
	White	6	19.36%
	Asian & Pacific Islander	1	3.23%
	Two or more races	7	22.58%
Sexual identity	Gay	22	70.97%
	Bisexual	3	9.68%
	Heterosexual	2	6.45%
	Non-gay or bisexual MSM	4	12.90%

Our findings can be summarized into three general thematic categories related to the process of becoming aware of PrEP and subsequent reactions it: hesitancy, neutrality, and partiality/enthusiasm. Hesitancy consisted of participants with mild to strong negative reactions to the concept of PrEP. Oftentimes, participants could not identify positive attributes of PrEP because they perceived no advantages, and sometimes perceived drawbacks to PrEP. Some, for example, expressed concern that PrEP would promote condomless sex. Those with hesitancy and negative perceptions about PrEP had to overcome these judgments in order to clear the way to regard PrEP as valuable and personally relevant. Participants who recollected overall neutral initial reactions to PrEP often had a general sense that it might be a good thing, but did not immediately perceive PrEP as a personally relevant prevention strategy. Immediate partiality/enthusiasm was rare in our sample—only one person reported this type of reaction upon hearing about PrEP, and took action to immediately seek a medical provider to gain access to PrEP.

### From Hearing to Learning About PrEP: Initial Exposure and Reactions to the Concept of PrEP

**Figure 1:**
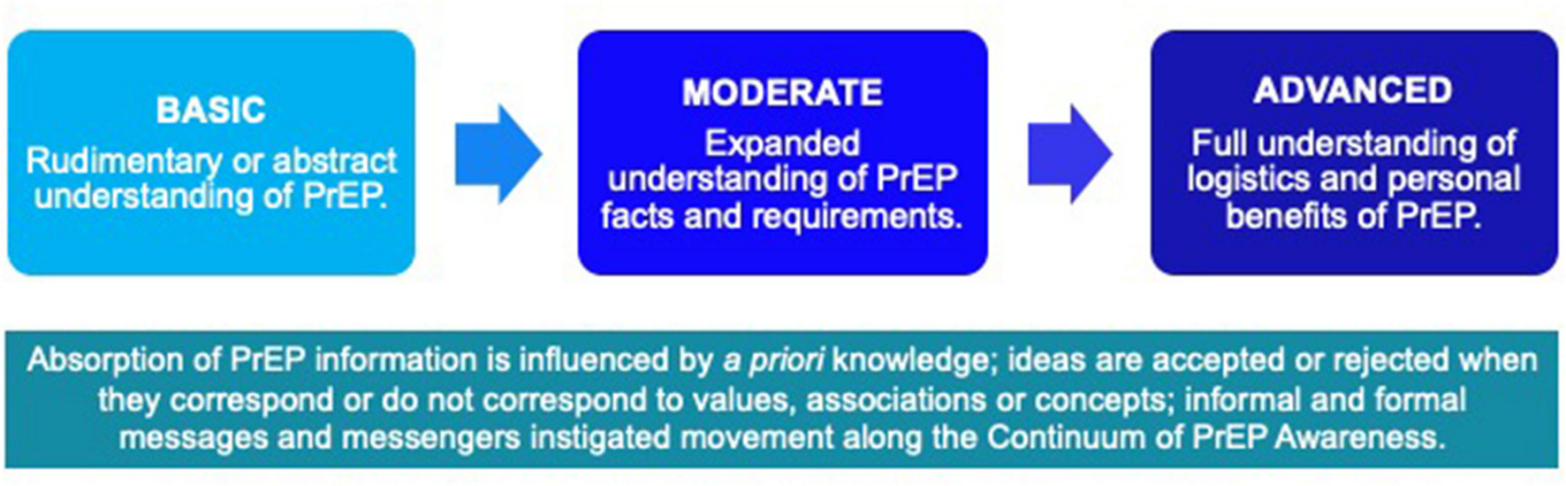
Continuum of PrEP awareness: from abstract to personally relevant.

We noted that awareness about PrEP or being “aware” of PrEP, meant a variety of things to our participants over different time periods suggesting that there are distinct phases associated with developing a consciousness about PrEP. The initial phase consisted of having a generic or basic grasp of the idea that a pill existed to prevent HIV infection. Next, awareness expanded to include a moderate understanding of PrEP facts e.g., it is a prescription medication, taken on a daily basis and requires on-going monitoring. In the advanced phase, awareness included seeing PrEP as personally relevant and beneficial (or if not personally relevant or beneficial, acceptance that it may be a good choice for others). Figure 1 depicts the continuum of PrEP awareness.

Participants reported initially hearing about PrEP through a wide range of direct and indirect communication channels, including exposure to news reports or hearing about PrEP directly from friends, roommates or family members. Many recollected early exposure to the concept of PrEP occurring on dating sites when individuals identified as “On PrEP” in their profiles. Others heard about PrEP for the first time from a health care provider or an HIV test counselor. Because the initial source of PrEP information varied widely, the content and messages about PrEP were equally varied in terms of quality and depth. For the vast majority of participants, it took a number of exposures to the notion of PrEP, usually through differing communication channels to fully absorb the concept of PrEP. We learned that for many there was a “spark” of interest after a single exposure to the concept, but not enough for participants to take active steps to seek out PrEP. This observation resonates with the widely accepted understanding of health behavior change—information alone is not enough to cause sustained and effective behavioral change.

### Key Influences on PrEP Awareness and Uptake Trajectories

Participants were fairly evenly split between those who were exposed to the concept of PrEP through formal, professional channels such as a primary care provider or an HIV test counselor and those who were exposed by non-professional peers, either friends, romantic partners, casual sex partners or family members. We noted a pattern among people who were exposed to the concept of PrEP by a healthcare provider or HIV or STI test counselor; these participants moved to PrEP uptake more rapidly than those who were exposed through other communication channels. Those who received information through non-professional channels expressed more ambivalence about the concept and remained in the moderate awareness phase for longer periods than those who were exposed through professionals. Messages about PrEP were seen as less authoritative and generated skepticism in some, but not all cases.

### Casual Sex Partners Build Basic Awareness: Slow to Warm Up

Miguel (all names are pseudonyms), a 20-year-old Latino gay man, was first exposed to the concept of PrEP during a visit to an HIV testing site. To our knowledge, the HIV test counselor did not provide him with information about PrEP. Instead, while in the waiting room he picked up a promotional brochure advertising the CRUSH Project and barely attended to the information as he reported that he was scanning it to “kill time”. He went on to describe his initial reaction to PrEP as “very irrelevant.” The concept of PrEP began to come into focus later on when a casual sex partner on PrEP mentioned it to Miguel in the context of explaining his preference for condomless sex. After this encounter, Miguel proactively sought out information about PrEP. He explained:

… he told me that he was on PrEP, and I was like, I don't know what it is. And, he said oh it's this pill called Truvada, you should look it up. Sure, but we're not having bareback tonight, just because I don't know what it is. So, I went home, I look it up, oh, fuck, it is a real thing, I thought he was making it up. So, I look it up and it rings a bell in my head, like, I heard that name before, I know I had. I didn't know where, and again, I forgot about it and then I saw the guy again and I told him, where'd you get your PrEP?

In Miguel's case, he expressed ambivalence and skepticism about PrEP because it was coming from a an unfamiliar and possibly unreliable information source. However, his encounter with this casual sex partner on PrEP motivated him to jumpstart the process of building PrEP awareness. This initial source that generated his interest was also the source that ultimately provided him with the essential information on how to access PrEP.

Like Miguel, Kevin, a 24-year-old Asian gay man, was slow to warm up to the concept of PrEP. Kevin described initial interest in, but no immediate action toward seeking PrEP after reading about it online. He attributed his reluctance to go on PrEP to concerns about his parents discovering his PrEP use. He also identified another critically important type of information that he was lacking, “I don't know how to do it.” In many cases, any early interest in PrEP was overshadowed by unknowns, uncertainties or incomplete information. Kevin's quote below highlights that learning about PrEP included learning about “how to do it:”

Interviewer: So here you are, you've read about PrEP. Then you finally decide to go on it, how did you make that decision?

Kevin: Right, so I should say that maybe there was a moment where in the back of my mind I was like yeah, it makes sense for me to do this. Like if it magically appeared I would do it, but then maybe it took an additional period of time for me to figure out the logistics of how do I get it? How do I pay for it? And will my parents know? And how do I avoid having that? So that was sort of a delay. And then the moment I got an HIV test from Oakland from a counselor–I asked about PrEP, because at the time I was more serious about trying to find it, he said he used to work at CRUSH and that I should check it out. …So yeah, there was this period where I was interested in it but not knowing specifically how to get it. So it was sort of in the back of my head.

Another participant, Thomas, a 23-year-old Latino gay man, reported something very similar in terms of having interest in PrEP, but not enough information or motivation to take it to the advanced phase of awareness:

I was kind of like, well, I guess I should get a doctor. But I never really made it past that because I didn't really know how to do that. I don't want my parents to know if I'm on it. I just felt like I had no idea how.

When a friend attempted to persuade a friend to consider PrEP, movement to advanced awareness and uptake was not usually immediate. For example, Garrett, a 25-year-old, Latino gay man recounted:

A friend of mine, he said, “You should take it.” I was like, “Uh, should I?” I wasn't sure if I really wanted to because you can just wear a condom. So after 2 months of thinking about it and talking with a few friends who were on PrEP I was like, hey, why not? He's just being extra safe and it's not going to cost me anything.

Interestingly, participants partnered with persons living with HIV shared a neutral, rather than an enthusiastic, response and like those cases depicted above were also slow to warm up to the concept of PrEP. One participant in particular, (Noah, a 28-year-old White gay man) had heard about, but had not pursued getting on PrEP until he was encouraged to do so by an HIV test counselor. Below he explains the timeline of learning about PrEP and the context in which he was prompted to pursue it:

… He and I had been together for a little while…and I decided that it was probably good for me to get tested... the person that tested me said that I should definitely look into the project here and see if it would benefit me. And so I came in about a month or two later and got signed up with the project... I had heard about [PrEP]. I didn't have as much knowledge at that time, but I'd definitely heard about it and it was something I was interested in getting more information about. And then when [test counselor] mentioned this–it made sense.

This particular participant belonged to the PrEP Facts Facebook group run by a US-based PrEP user and advocate. He noted that the site served as a useful, convenient and supportive community to turn to for information about PrEP. Importantly, he described the information as making “*more* sense” after he initiated PrEP use: “it definitely made a lot more sense to read it after I was on PrEP.” This demonstrates how the same information can be processed differently by a PrEP naïve persons versus by someone with personal PrEP experience.

### Providers as Authoritative Messengers Motivate or Obstruct PrEP Uptake

In situations where a medical provider or an HIV test counselor introduced a patient/client to the concept of PrEP, the message often sunk in more readily than for those who were exposed through other means. In many cases, medical providers were perceived as a reliable source of information about PrEP (because PrEP fit within the medical technology paradigm) and this stature afforded to providers meant that they could either persuade or dissuade patients from utilizing PrEP. We noted that providers often served as a key steppingstone to facilitate absorption of PrEP information at the moderate level and, in several instances, offered a message that prompted patients to see themselves as viable PrEP candidates for the first time. For example, Christian, a 26-year-old Latino gay man asked his “very knowledgeable Kaiser doctor” about whether he thought people should be on PrEP. His provider apparently responded “Yes” with “no question in his voice.” This unequivocal endorsement of the value of PrEP from a trusted authority sufficiently motivated Christian to join the CRUSH study as a PrEP user. Another young Latino gay man, age 25, was told by his provider “You're a great candidate for PrEP. You should start using it.”

Sometimes when participants associated PrEP with “unsafe” sexual practices, the authority of a medical provider turned around these negative associations with PrEP. This was the case explained to us by Thomas, a 23-year-old Latino gay man:

It was coming into a hospital, people who I saw as authorities, saying that this is a safe thing to do. My experience before was that [just] a dude was telling me this. He was saying like, “oh, PrEP is a medication to prevent HIV.” And I'm like, “OK, but no. What does that mean?” … [CRUSH clinic] setting–I could see people who I saw as credible authorities. So, instantly I was just like, “OK, this is legit.” That's really what it was. “OK, I trust you..”..I flipped, yeah, completely, because [before CRUSH], I was not for it. I was like, “I don't trust PrEP. I don't know what the fuck that is.”

Michael, a 26-year-old Latino gay man, like many of our participants, was first exposed to the concept of PrEP while on a dating app. He initially found the concept to be interesting, but not necessarily directly relevant. He reported that he did not “immediately talk to his doctor about it.” It is possible that Michael's statement implies that speaking to his doctor would be a logical next step.

I was out of a relationship and had Grindr and there were all these profiles of, like, “Neg on PrEP.” And I was like, “PrEP? What's PrEP?” So, I did just look up “PrEP.” At first, it was still very new for me and I didn't jump on it right away. It was interesting. I mean, it was definitely a great thing to be able to be safer around, like, your sexual health and doing an extra thing to ensure that you would not get HIV at the time. And even after I first learned about it, I didn't immediately talk to my doctor about it. I actually talked to one of my friends about it. He was working doing, like, HIV outreach. And he's like, “Yeah, like, it's great. It's not for everyone but, you know, like, it seems like it really works.” And he answered a lot of questions for me.

Interestingly, when Michael approached his doctor requesting more information about PrEP, his doctor purportedly said “Are you sure this is something you want to consider?” which caused him to “second guess” his decision. This second guessing stopped him from pursuing going on PrEP and it was a few months later when he returned to his provider and told her that “I did want to get a referral” and she then referred him to the CRUSH project.

In addition to Michael, a handful of participants reported unsupportive comments made by their primary care providers about PrEP e.g., “you're not a prostitute or something?” Other providers outright refused to prescribe PrEP as was the case for Tyler, a 25-year-old White gay man:

I don't know how many other clients she has that are gay, but she was always asking me how many people have you slept with... She makes me seem very skanky…. she's the one that wouldn't give me PrEP….. it was kind of disappointing, because I had to work up the courage to make an appointment, and then ask her, and then for her to, like, say, well, I don't know what it is, and then she wouldn't prescribe it.

### Positively Framed PrEP Messages Motivate Shift From Moderate to Advanced Awareness and Uptake

While the combination of the learning context, the PrEP messenger and the message were highly impactful, we noted that the content of certain messages were regarded as potent influences on moving people along the continuum toward PrEP adoption. Thematically speaking these messages explicitly conveyed the idea that *PrEP use is sensible*. Implied in some of the *PrEP use is sensible* messages was a regard for sexual wellness, a broad concept that encompasses notions of sexual safety, self-determination in one's sex life, and comfort with sexuality. Positively framed messages about PrEP delivered during opportune moments allowed some participants to orient or reorient themselves to see PrEP as a sensible choice. For example, Garrett moved from uncertain about whether he ought to use PrEP to “why not” when he told himself that his friend was taking it to be “extra safe” and that this safety was a compelling reason to use PrEP. Or when Gabe's medical provider suggested that PrEP could be considered a back-up method of HIV prevention, rather than a primary line of defense. This particular message allowed Gabe, a 27-year-old Latino gay man, to see himself as good at preventing HIV, but not infallible:

He said it looks like you don't need it as much but even just one time can provoke disease to come in. So, having PrEP would help you avoid, even if you had those slips. You know?

Colin, a 26-year-old Black bisexual man, related hearing a similar message that helped him to see PrEP as personally relevant. In this case, the HIV test counselor leveraged Colin's expressions of concern about his vulnerability to HIV:

He just told me that if I feel like I'm having that much sex and I need to protect myself, that this was something that could help better protect me. He didn't tell me it was going to be a lifesaver, but he was just like, it'll better protect you.

For Jeremy, a 24-year-old White gay man, he pinpointed the message that shifted his attitude toward PrEP stating, “what struck me was when someone compared it to the contraceptive.” This analogy allowed Jeremy to change his associations with PrEP use, from a negative “slut shaming” message, to seeing PrEP as an assertion of a human right–“the right to your own body and health.”

And in the case of Kenneth, a 22-year-old Black gay man, he responded to the idea of using PrEP as a source of “extra” protection–prior to that he could not see PrEP as relevant to his life as a consistent condom user. The provider's message helped to mitigate negative feelings:

…she's like, it's just that extra protection. You know, and she really stressed that to me. It's just that extra protection that will be helpful, especially when you go to get tested. It will alleviate some of those butterflies that you get when you're not sure [about the] result. And, that, for me, when she said that, basically like, it sold it for me.

For those with hesitant or negative perceptions, having a messenger perceived to have some authority such as an HIV test counselor or a medical provider, increased their willingness to change their opinion or accept science-based messages about PrEP. Though in underscoring our main finding, even these persuasive messengers and messages did not produce a significant effect if they *introduced* the concept to a PrEP naïve client, patient, friend. Returning to Colin's case, although he frequently tested for HIV and was counseled to consider PrEP while testing, it took three interactions with the same test counselor before he eventually “got it”—the concept of PrEP and its potential relevance to his life. He explained:

... one day, he was just like, “You come in here a lot. What's up?” I'm just like, “I do a lot and I'm scared. I don't want anything to happen. I want to at least know if I do have something” He was like, maybe you should look into PrEP... I took the packet, but I didn't think too much about it because I didn't know what it [PrEP] was and didn't read it. I just kind of tossed it aside. And, I came back about two more times and he was just like, “oh, you're still here–you never looked at that packet I gave you.”

After the third encounter with an HIV test counselor encouraging him to seek out PrEP, Colin contacted the CRUSH PrEP navigator and initiated PrEP use a few days later. He went on to become an informal advocate of PrEP and the CRUSH study among his extensive social and sexual networks.

## Discussion

In this analysis, we described the processes by which PrEP users came to learn about, eventually digest information about, and subsequently adopt this HIV prevention modality. Our participants usefully reflected on their initial reactions and the subsequent events leading up to their eventual uptake of PrEP. These reflections directed us to carefully consider communication channels, messages and messengers. We noted the different influences associated with the two most common communication channels by which young men were initially exposed to the concept of PrEP: formal/professional and informal channels.

After initially digesting the concept of PrEP, participants moved to an increased level of awareness or a cognitive activation process of deepening one's knowledge and interest in PrEP. We call this the *advanced* or *awakened* phase of PrEP awareness. Our analysis led us to interpret this phase not in the classic terms of retaining facts about PrEP, rather we defined it as the moment in which PrEP became personally salient for men. Indeed, this moment was not divorced from facts—facts were necessary, but insufficient for PrEP uptake. PrEP uptake happened when the combination of facts and a shift in self-perception occurred which then allowed PrEP to be considered in a new light. We noted that the “real” learning about PrEP often took place during times when people were in a situation or frame of mind when they understood themselves to be vulnerable to HIV—i.e., when seeking testing and/or treatment for a sexually transmitted infection or testing for HIV after high-risk sexual contact.

Often, the initial exposure to the concept of PrEP prompted participants to further investigate the topic. For others, the initial exposure was not impressive enough to warrant further investigation—we labeled this reaction as akin to the human information processing theory of “shallow processing” (32). It was not until they encountered PrEP information repeatedly in future contexts that they began to more fully attend to and absorb the concept of PrEP. Uptake of PrEP took time whether participants were initially interested in finding out more about PrEP or not. Participants rarely reported becoming well-informed about PrEP over the course of an initial exposure. Rather, learning occurred after multiple exposures to PrEP information through numerous intersecting forms, messengers and communication channels namely social media, healthcare professionals, HIV test counselors, family members, friends, peers and PrEP users.

Hearing about PrEP certainly did not confer full understanding of it, and our analysis demonstrates additional events that must transpire before PrEP uptake occurs. To achieve PrEP utilization at the level necessary to truly impact the HIV epidemic, efforts to deliver salient messages by influential messengers must be scaled up. The usual marketing channels such as social marketing and advertising are important, but our findings suggest that there are communication channels that could be better leveraged such as providers, HIV test counselors and PrEP users themselves.

We posit that there is a *continuum* of PrEP awareness that can and does promote PrEP learning by allowing formal and informal PrEP educators to understand where an individual is along the continuum and to tailor a message according to that phase. This can allow the educator to adjust one's expectations about when and if PrEP uptake might occur. Building demand among those in the basic awareness phase will take longer than those in the moderate phase. Many young, gay and bi-sexual male prospective PrEP users will benefit from messages that promote PrEP as a tool to promote individual- as well as community-level sexual wellness. Leveraging messages that emphasize personal benefits, including social, sexual and emotional benefits can be persuasive. More concretely, if a patient is handed information about PrEP in a medical office, delivering it with a tailored message and then encouragement to seek out online videos to hear personal stories could be a powerful educational strategy.

Our paper makes a novel contribution to the literature by promoting the concept of a continuum of PrEP awareness. We have broken down the phenomenon of PrEP awareness into three phases: basic, moderate and advanced. Disentangling the distinct ways in which individuals are “aware” of PrEP serves as a useful starting point to determine how to proceed with client-centered PrEP education and promotion strategies. Our findings illustrate that primary care providers can facilitate or inhibit PrEP uptake. We encourage primary care providers to carefully handle their power to persuade by avoiding micro-aggressions, off-hand and dismissive comments such as “are you sure you need it?” Providers may consider these types of comments as minor challenges to a patient and unintentionally undermine the fragile patient-provider relationship. Instead, providers need to be educated on the importance of the collateral or social and emotional benefits conferred by PrEP (33). In addition to the biological benefits of PrEP use, the benefits associated with feelings of safety and empowerment can and should be emphasized by formal PrEP messengers. Thus, we recommend training formal messengers e.g., HIV test counselors and primary care providers serving gay and bisexual men, to ask clients and patients a simple question: Did you know that there is a pill to prevent HIV? If they do not, then the messenger can provide basic PrEP facts e.g., PrEP is a prescription medication that is highly effective at preventing HIV; it has minimal side effects and is typically covered by public and private insurance plans. In addition, our research demonstrates the power of positive, pragmatic messages about PrEP that make it directly relevant to the person at risk, for example a brief description of how PrEP has made a difference in the lives of other young, gay and bisexual men. These brief messages portray PrEP as a commonsense HIV prevention strategy and might be framed in the following ways: *using PrEP can provide a sense of control over your sex life and your sexual health*; *using PrEP can help decrease anxiety or fear related to HIV*; *using PrEP can help by taking something off of your “worry list.”* PrEP messages that allow individuals to tangibly anticipate how their lives may be improved by using PrEP can create this sense of personal relevance. Once individuals perceive that PrEP is personally relevant, uptake may be the next step.

Our research is limited by the homogeneity of our sample; each participant had a history of PrEP use and had been exposed to numerous messages about PrEP through their participation in the demonstration project. Because we asked participants to describe their initial reaction to PrEP upon first hearing about it, we recognize that the timeline introduces a potential for bias and may have colored how initial reactions were reported. One strategy we used to ensure that our interpretations of these learning narratives were not off base, was to share these findings in the context of a community forum that consisted of many of our research participants, as well as members of the gay and bisexual men community that were interested in the topics discussed during the forum. Feedback during and after the forum was overwhelmingly positive. Further, participation in the CRUSH demonstration project allowed study participants to access PrEP medication, laboratory and clinic visits for free. This removed the known barrier of cost. Concerns about cost of accessing PrEP can be even more acute for young men who may be unwilling to access PrEP if they rely on a parent's insurance plan. Our findings must be considered in light of these factors. While producing generalizable findings is never the goal of qualitative research, we hope that our findings will have resonance and that there may be transferability of these insights to similar populations (31).

This study allowed us to illustrate how a sample of young gay and bisexual men learned about and made the decision to use PrEP. This continuum of PrEP awareness depicts how participants moved from *hearing about* to *learning about* to *utilizing* PrEP. Moving from PrEP awareness to uptake required a multi-step processing of exposure to information, digesting the information and identifying the technology as personally relevant. Importantly, our findings shed light on the conditions under which participants moved from awareness to uptake. Encouragement from a medical professional or an HIV test counselor persuaded some youth to quickly adopt PrEP, whereas it took longer for others to adopt PrEP who were exposed to PrEP information through more informal communication channels such as friends and prospective sexual and romantic partners. These findings are significant because they allow us to understand how our participants responded to formal and informal communication about PrEP and how this awareness influenced PrEP uptake. Organizations and individuals working to improve PrEP awareness and uptake may find value in use of this continuum.

## Data Availability Statement

The datasets presented in this article are not readily available because we do not have IRB approval to share data beyond the immediate study team. Requests to access the datasets should be directed to kimberly.koester@ucsf.edu.

## Ethics Statement

The studies involving human participants were reviewed and approved by University of California, San Francisco and Sutter Health Institutional Review Boards. The patients/participants provided their written informed consent to participate in this study.

## Author Contributions

KK: conceptualized study design, methods and manuscript development with substantial contribution from XE to methods, and intellectual development of manuscript. IU and JM: made substantial contributions to the study concept and design and intellectual content. JB and M-SK: contributed to study development and revised the article for intellectual content. All authors have approved the content and agree to be accountable for the work.

## Funding

This research was support by the California HIV/AIDS Research Program.

## Conflict of Interest

The authors declare that the research was conducted in the absence of any commercial or financial relationships that could be construed as a potential conflict of interest. The reviewer JB declared a shared affiliation with the authors to the handling editor at the time of review.

## Publisher's Note

All claims expressed in this article are solely those of the authors and do not necessarily represent those of their affiliated organizations, or those of the publisher, the editors and the reviewers. Any product that may be evaluated in this article, or claim that may be made by its manufacturer, is not guaranteed or endorsed by the publisher.
